# Trauma, empowerment, and resilience: understanding the mental health concerns of people living with human immunodeficiency virus in Manitoba

**DOI:** 10.3389/fpubh.2026.1733501

**Published:** 2026-02-10

**Authors:** Adi Keynan, Enrique Villacis-Alvarez, Katharina Maier, Margaret Haworth-Brockman, Joel Baliddawa, Freda Woodhouse, Kyda Archie, Marj Schenkels, Nikki Daniels, Rebecca Murdock, Robert Russell, Lisa Patrick, Yoav Keynan, Lauren MacKenzie, Laurie Ireland, Ken Kasper, Zulma Vanessa Rueda, Beverley K. Fredborg

**Affiliations:** 1 Department of Psychology, The University of Winnipeg, Winnipeg, MB, Canada; 2 Department of Medical Microbiology and Infectious Diseases, Rady Faculty of Health Sciences, University of Manitoba, Winnipeg, MB, Canada; 3 Department of Criminal Justice, The University of Winnipeg, Winnipeg, MB, Canada; 4 College of Community and Global Health, Rady Faculty of Health Sciences, University of Manitoba, Winnipeg, MB, Canada; 5 National Collaborating Centre for Infectious Diseases, Rady Faculty of Health Sciences, University of Manitoba, Winnipeg, MB, Canada; 6 Peer Research Team, AllTogether4IDEAS Research Consortia, Winnipeg, MB, Canada; 7 Department of Internal Medicine, Rady Faculty of Health Sciences, University of Manitoba, Winnipeg, MB, Canada; 8 The Manitoba HIV Program, Winnipeg, MB, Canada; 9 Nine Circles Community Health Centre, Winnipeg, MB, Canada; 10 Department of Family Medicine, Rady Faculty of Health Sciences, University of Manitoba, Winnipeg, MB, Canada; 11 Health Sciences Centre, Shared Health, Winnipeg, MB, Canada; 12 Escuela de Ciencias de la Salud, Universidad Pontificia Bolivariana, Medellin, Colombia

**Keywords:** childhood trauma, empowerment, engagement in HIV care, human immunodeficiency virus, Manitoba, mental health, qualitative study, resilience

## Abstract

**Introduction:**

Canada has endorsed the Joint United Nations Programme on HIV/AIDS (UNAIDS) target to end the HIV epidemic by 2030. However, Canada has reported increases in HIV diagnoses for five consecutive years since 2019, with Manitoba’s provincial rates three times the national average. This study examines mental health factors that may impact engagement in care among people living with HIV in Manitoba.

**Methods:**

For this qualitative study, 32 Manitoban women, men and gender-diverse people living with HIV completed semi-structured interviews on barriers and facilitators to HIV care; as well, we incorporated quantitative measures on childhood trauma and empowerment. Thematic analysis revealed four themes: experiences of hardship, substance use as coping, resilience, and empowerment.

**Findings:**

Most participants reported experiencing numerous hardships, including severe childhood trauma, interpersonal violence, HIV stigma, racism, and socioeconomic-related stressors. These adversities likely synergistically exacerbate participants’ current mental health concerns, undermining HIV treatment engagement and adherence. Despite these challenges, many participants described gaining empowerment and demonstrated resilience through health-promoting behaviors (e.g., enlisting social supports). A key sentiment among participants was that while they feel supported by their HIV care providers, they require additional supports for substance use, mental health, and social determinants of health such as housing, which interfere with engagement in HIV care providers, they require additional supports that address key social determinants of health, including substance use, precarious housing, and mental health support to address traumatic experiences, as these factors interfere with engagement in HIV care.

**Conclusion:**

The diverse experiences of participants may worsen mental health and hinder HIV treatment engagement. However, simultaneously, participants demonstrated resilience and empowerment in their daily lives. Future research should focus on strengthening resilience, empowerment, and mental health to improve outcomes for PLHIV in Manitoba.

**Interpretation:**

To achieve the UNAIDS targets, it is essential to conduct research and implement evidence-based mental health interventions, along with related strategies that foster resilience and empowerment in this population.

## 1 Introduction

Human immunodeficiency virus (HIV), a sexually transmitted and blood-borne infection (STBBI), remains a significant health concern internationally, despite achievements in prevention, diagnosis, and treatment. In 2024 alone, an estimated 40.8 million people were living with HIV, with 1.3 million new infections, and 630,000 HIV-related deaths reported worldwide (1). These statistics underscore the urgent need for sustained action and improved patient outcomes.

To improve efforts in reducing HIV worldwide, the Joint United Nations Programme on HIV/AIDS (UNAIDS) established global targets to reduce new HIV infections by 90% to “end the AIDS (acquired immunodeficiency syndrome) epidemic” by 2030. To achieve this, UNAIDS initially proposed a “90–90-90” goal, and in 2020, this shifted to a “95–95-95” target set for 2030, with the objective of improving health outcomes and decreasing HIV transmission. This means that by 2030, the goal is to diagnose 95% of all people living with HIV (PLHIV), for 95% of people diagnosed with HIV to receive ongoing antiretroviral therapy (ART) treatment, and for 95% of those receiving ART to achieve viral suppression (1).

Despite its status as a high-income country with universal, publicly funded health care and ART in many provinces, Canada is currently in the midst of an HIV epidemic. As of 2022, 65,270 Canadians were living with diagnosed HIV, and while some progress has been made towards the UNAIDS targets, an estimated 10% of PLHIV are still unaware of their diagnosis, and at least 15% of people diagnosed with HIV are not taking ART (2). In addition, new infections have been increasing nationally making the reduction of new infections by 90% by 2030 unlikely to be met nation-wide. In the province of Manitoba, there has been a steady increase in new HIV diagnoses over the past 8 years, including a 48% rise in cases from 2017–2022, surpassing the national average threefold (2). Between 2018 and 2021, 81.2% of newly diagnosed PLHIV in Manitoba began ART, and of those individuals only 64·9% achieved an undetectable viral load (2). Manitoba’s HIV incidence differs from national trends, with a disproportionate impact on young people, females, people who inject methamphetamine, heterosexual and cis-gendered individuals, and Indigenous peoples (2). Furthermore, Manitoba is one of the *few* provinces to have completely missed the UNAIDS target in 2020, with the main contributing factors considered to be a confluence of *syndemics* (2).

Singer’s syndemic theory posits that interactions between coexistent diseases exacerbate each disease’s burden on the individual, acknowledging that a person’s health is dependent on multiple interacting layers (3). In Manitoba the syndemic conditions seen in people newly diagnosed with HIV include injection drug use, houselessness, and mental health challenges (2).

Previous research in Manitoba has reported PLHIV’s perspectives on barriers and facilitators to accessing HIV care (4). Mental health challenges emerged frequently as a barrier, which included stigma and psychological distress stemming from unresolved trauma, including intergenerational trauma, that predated their HIV diagnosis. Mental health research in PLHIV has additionally found substance use to have negative effects on ART continuation (5, 6).

In addition, empowerment of PLHIV may play a critical role in engagement in HIV care. Individual patient empowerment in health care is “a process that enables patients to exert more influence over their individual health by increasing their capacities to gain more control over issues they themselves define as important” (7), (p. 1927). Empowerment can be an *outcome*, meaning that empowerment lies not solely in the ability to make decisions and control one’s life in the here-and-now, but also in a person’s sense of increased autonomy over time.

Research on trauma, health care, and empowerment has highlighted that previous and/or current experiences of trauma may impact a person’s levels of empowerment (7), and that individuals living with chronic illness who are less empowered tend to have worse health outcomes (8). Literature examining medical outcomes among PLHIV found empowerment to be positively linked to medication adherence, disease management, and general self-efficacy (9). Given that many PLHIV have experiences of childhood trauma, and that individuals who have experienced childhood trauma tend to lack important protective factors (e.g., social support) and often report reduced levels of empowerment (10), understanding how to improve empowerment in PLHIV in Manitoba can improve treatment outcomes.

The findings that mental health symptoms, such as posttraumatic stress symptoms, influence HIV treatment adherence underscore the importance of taking an intersectional lens. Intersectionality theorists are concerned with how intersections of gender (e.g., identity roles, relational power), sex at birth, race, age, and other factors influence the lives of individuals and populations. Intersectionality reveals how combinations of unique social identities can result in varying forms of privilege or discrimination (11), influencing care experiences, levels of HIV-related stigma, and mental health (5). For example, in cases where an individual is struggling with housing alongside HIV, a hierarchy of urgent needs tends to form resulting in the de-prioritization of HIV care. As such, new standards of care have been developed, including *trauma-informed care*, an organization-level intervention that centers on elements of systems, structures, and service delivery to serve clients with a trauma history (12). Such efforts to reduce barriers to care are crucial considering that a significant proportion of PLHIV in Manitoba concurrently face challenges such as substance use and poverty (2, 11).

To provide a novel mental health perspective to the HIV literature, we investigated the experiences and relationships of mental health variables, empowerment, substance use, trauma-related experiences, and resilience among PLHIV in Manitoba. We had three primary objectives (1) highlighting the experiences of Manitobans living with HIV, (2) understanding why Manitoba is witnessing an increase in the HIV trends relating to syndemics, and (3) establishing a foundation for evidence-based mental health interventions for PLHIV.

## 2 Methods

### 2.1 Community engagement

This study used a participatory action research design which relies on, emphasizes, and collaborates with people who have lived/living experiences to produce new knowledge and translate it into action. We fostered participant collaboration, maintaining strong relationships with community members and organizations throughout the process, and ensured that the findings were both informed *by* and beneficial *to* the community (2, 13). We maintained a collaborative approach throughout the larger project by forming a Research Advisory Committee (RAC) and a Peer Research Team (PRT) (2). Given that Indigenous people and women are being disproportionately affected by new cases of HIV in Manitoba, the RAC comprised an Indigenous elder; Indigenous leaders; community members, many of whom are women and are Indigenous, with lived and living experience with HIV, substance use, houselessness, and/or intergenerational trauma; and researchers and academics specializing in HIV, STBBI, and sex- and gender-based analyses. The PRT also included community members who have lived/living experiences of HIV, substance use, and/or houselessness. Throughout these projects, the PRT met bi-weekly to exchange skills and knowledge, while also organizing and participating in a range of activities including outreach initiatives, resume building, professional development, and engagement in general health and advocacy presentations, both as attendees and presenters. We also collaborated with numerous community partners (e.g., Nine Circles Community Health Centre in Winnipeg, Manitoba), ensuring that the voices of PLHIV and people who inject drugs (PWID) were included.

### 2.2 Materials

This qualitative study involved re-analyzing data from semi-structured interviews, comprising 26 exploratory questions co-designed with PLHIV in Manitoba, from a mental health perspective (13). The primary purpose of the interview was to understand how PLHIV interact with and perceive health care services, with a particular focus on factors that facilitate or hinder their engagement. Questions focused on topics such as the participants’ HIV journey regarding their diagnosis, including how different social factors influenced their adherence to treatment (e.g., substance use, socioeconomic status), and their suggested improvements to HIV care. We re-analyzed the data for the current study as many individuals spontaneously mentioned mental health concerns and needs throughout their interviews, which were not captured in the first study, as this was focused on the barriers and facilitators to treatment engagement.

Participants also completed three self-report quantitative questionnaires:

#### 2.2.1 The socio demographic and life circumstances questionnaire

The participant socio demographic and life circumstances questionnaire (13) collected data on participants’ health practices (e.g., harm reduction, health knowledge, and substance use) and structural factors (e.g., unstable housing, poverty) that may impact their health.

#### 2.2.2 Childhood trauma questionnaire-short form

The CTQ-SF (14) is a self-report measure comprising 28-items meant to categorize participants’ early life traumatic experiences into five subscales (i.e., emotional abuse, physical abuse, sexual abuse, emotional neglect, physical neglect) ranked by their severity (i.e., none, low, moderate, severe). Items are rated on a 5-point Likert scale ranging from 1 (“never true”) to 5 (“very often true”) and summed to form a total score and the respective subscale scores. The internal consistency for this study’s total CTQ-SF was excellent (Cronbach’s *α* = 0·90).

#### 2.2.3 Empowerment scale

We used the 25-item version of the Empower-Making Decisions survey (15). This measure provides participants with an intrapersonal empowerment score and comprises five factors: self-esteem-self-efficacy, power-powerlessness, community activism and autonomy, optimism and control over the future, and righteous anger. Items are rated on a 4-point Likert scale ranging from 1 (“strongly agree”) to 4 (“strongly disagree”) and empowerment was measured using the mean of the summed total score. This study’s internal consistency for the Empower-Making Decisions survey was acceptable (Cronbach’s *α* = 0.76).

### 2.3 Setting

The province of Manitoba is located at the longitudinal centre of Canada. In 2021, Manitoba was home to approximately 1.35 million people, with most of the population residing in the cities of Winnipeg and Brandon. Any person who is diagnosed with HIV in the province is referred to the Manitoba HIV Program, which actively provides treatment to over 2,500 PLHIV across seven sites (16). This study took place across three of the Manitoba HIV program center’s (i.e., one clinic located in Brandon, and two clinics in Winnipeg).

### 2.4 Participants

The full protocol of the study was published elsewhere (13). To participate in the study, individuals had to be at least 18 years old, have an HIV diagnosis, and reside in Manitoba. Participants were recruited at the Manitoba HIV clinics and received referrals from HIV service providers. Advertisements via social media and posters in HIV clinics were also employed. Participants were compensated $50 CAD for their time. Once thematic saturation was reached, qualitative data sampling halted.

This study was approved by the University of Manitoba Health Ethics Research Board (HS25572; H2022:218), as well as the First Nations Health and Social Secretariat of Manitoba, Nine Circles Community Health Centre, Shared Health Manitoba (SH2022:194) and 7th Street Health Access Centre.

Thirty-four participants completed qualitative interviews, with 32 completing the CTQ-SF and 31 completing the empowerment measures surveys, respectively. Two participants were excluded because they did not have an HIV diagnosis, and one participant fell ill during the self-report portion of the study visit.

This article analyzed the 32 participants who completed the qualitative interviews and met none of the exclusion criteria (*M*_age_ = 43.31, *SD*_age_ = 11.75; 31.3% cis-women, 56.3% cis-men, 3.1% Non-Binary, 6.2% Two-Spirit) using purposive/selective sampling methods, with the intention of prioritizing Manitobans with recent HIV diagnoses. Most participants (59.40%) identified as Indigenous, 12.50% as Southeast Asian, 12.50% as White/European, and 15.63% as ‘other race’ (2, 4, 13–15).

### 2.5 Procedure

Data collection occurred between October 2022 and May 2023 in a private room to ensure privacy and confidentiality. Following the informed consent process, a semi-structured qualitative interview took between one and two-and-a-half hours, depending on the extent of participant sharing and allowing for the flexibility for participants to take breaks if needed. Following the interview, participants completed three paper-and-pencil self-report questionnaires.

The interviewer (EV) was trained in qualitative research and interviews, had no pre-existing relationships with the participants, and was not involved in the proposal of the project, to reduce potential bias. The interviewer was accompanied on-site by the team’s Indigenous cultural advisor and peer co-researchers to support participants during breaks. In preparation for the interview, participants could request the presence of peer co-researchers to attend their interview, or to talk with counsellors for emotional support.

### 2.6 Data analysis

#### 2.6.1 Data preparation

All interviews were audio-recorded and then transcribed using the Otter.ai (Inc.) tool. Transcripts were reviewed by EV for accuracy.

#### 2.6.2 Analysis procedures

For quantitative results, we used SPSS Statistics Version 29 for descriptive analyses (17). To analyze the qualitative data for this study, we used NVivo (R) 15 Pro. Our qualitative analysis was informed by Braun and Clarke’s iterative six-step guide to thematic analysis (17, 18). The initial codebook was drafted by researchers familiar with the data along with the use of a deductive approach. For this step, following data familiarization, AK and BF deliberated potential parent and child codes for the initial codebook. Next, AK completed open coding on five transcripts. This step involved the consolidation of themes, collapsing and separating them as necessary. This resulted in modifications and additions made to the codebook, which were reviewed by and created in collaboration with BF and ZR (trained researchers in the fields of clinical psychology and HIV, respectively). The final step of codebook drafting involved a literature search for mental health themes commonly used in the HIV and/or injection drug use context to ensure that no codes or definitions were neglected. Once all codes were clearly defined and agreed upon by AK, BF, and ZR, AK completed coding for the remaining transcripts, while documenting any relevant comments or discrepancies that arose. To maintain the iterative process in the thematic analysis during the coding of remaining transcripts, some additional themes were added to the codebook that were not adequately captured previously.

**Figure 1:**
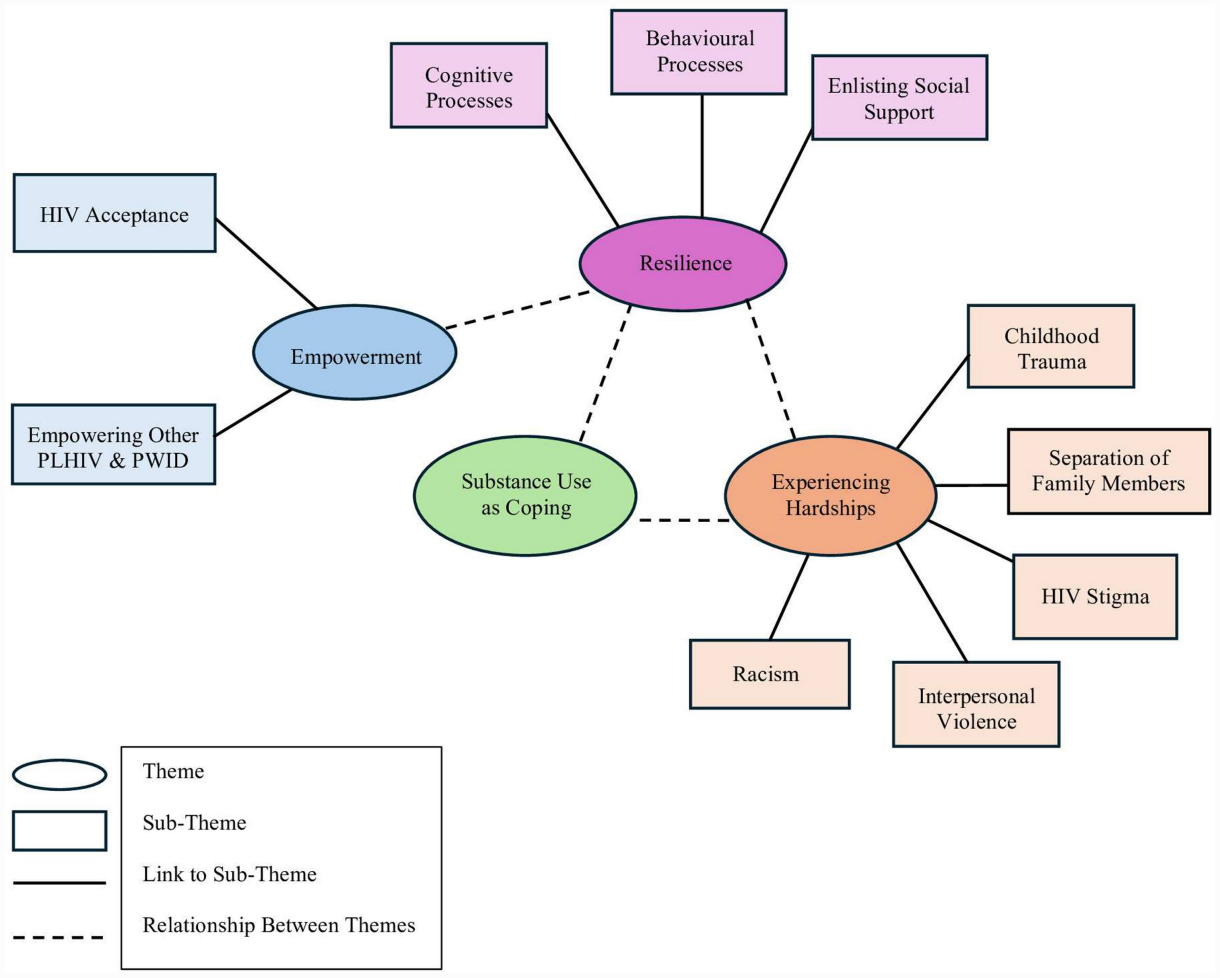
Thematic map of the four main themes and sub-themes reported by participants living with HIV in Manitoba.

Throughout the entire analysis process, the three researchers (BF, ZR, and AK) engaged in reflexive research collaboration to maintain open communication in the discussion of themes (19). Doing so prioritized the credibility of themes, ensuring they accurately represent the data (17, 20). Once coding was completed, researchers drafted the thematic map (Figure 1) and developed four themes: experiences of hardship, substance use as a coping mechanism, resilience, and empowerment. We defined *empowerment* as both a *process* (i.e., gaining power to influence environment) and an *outcome* (i.e., resulting in solutions) (21).

While there are numerous, disputed definitions of resilience, in this article we have adhered to the *multi-system model of resilience* (MSMR) which complements several existing definitions (22). The MSMR defines resilience as a person’s capacity to operate across a continuum, ranging from vulnerability to resilience. It posits that a person’s place on the continuum is dependent on a reservoir of internal and external resources, as well as their coping mechanisms, in response to their needs and situation. To operationalize resilience, we have selected two primary thematic areas (i.e., intrapersonal and interpersonal resiliency) adopted from Harper and colleagues’ research on resilience in PLHIV (6).

Given that Manitoba has witnessed higher HIV incidence rates among women, it is essential to understand sex and gender related experiences. We report data by sex at birth and gender to understand what unique social determinants of health (e.g., intimate partner violence, childcare duties) are experienced by women living with HIV. Lastly, the theoretical underpinnings of our research are influenced by Singer’s syndemic theory (3). Our findings include representative quotes from participants.

## 3 Results

### 3.1 Empowerment scale

The total score of the Empower-Making Decisions scale for women (mean = 1.94 and 0.34) was lower than for men (2.04 ± 0.23) (Table 1).

**Table 1:** Participants living with HIV and childhood trauma questionnaire-short form and empowerment scores.

Variable	Female (*n* = 10)		Male (*n* = 21)		Total (*n* = 32)*	
	Median	IQR	Median	IQR	Median	IQR
CTQ total	73.5	44.75–97.5	63	47.5–83	65	47.25–83
CTQ sexual abuse	16.50	5–21.75	12	5–18	13	5–19.75
CTQ physical abuse	9	5–17.25	9	7–14.5	9	7–16.5
CTQ emotional abuse	15.50	9–18.25	11	7–16	12	7.25–16.75
CTQ physical neglect	12	6.5–16.25	10	6–13	11	6.25–13
CTQ emotional neglect	12.50	9.75–17.25	12	8.5–17	11.5	9–16.75
CTQ minimization and denial	7	5–9.25	8	5.5–11	7.5	5.25–10

Variable	Female (*n* = 10)		Male (*n* = 20)		Total (*n* = 31)	
	Mean	SD	Mean	SD	Mean	SD
Empowerment total	1.94	0.34	2.04	0.23	2.01	0.27

SD, standard deviation; IQR, Interquartile range; CTQ, childhood trauma questionnaire.

^*^Total has one additional participant because they selected not to identify their sex at birth.

### 3.2 Childhood trauma questionnaire-short form

Eighty-four percent of participants experienced at least one form of abuse. Of all forms of child abuse experienced, sexual abuse was most common, with 17 of 32 participants (53.13%) self-reporting a *severe* experience of this abuse. Participants also endorsed experiencing *severe* forms of physical abuse (40.63%), emotional abuse (34.40%), physical neglect (34.40%), and emotional neglect (21.90%) (Table 2).

**Table 2:** Participants living with HIV and childhood trauma questionnaire-short form severity.

Variable	Female		Male		Total (*n* = 32)	
	Frequency	%	Frequency	%	Frequency	%
CTQ total						
None	0	0	0	0	0	0
Low	3	9.40	7	21.90	10	31.30
Moderate	2	6.30	5	15.63	7	21.90
Severe	5	15.63	9	28.1	15	46.9
CTQ sexual abuse						
None	3	9.40	7	21.90	10	31.30
Low	0	0	3	9.40	3	9.40
Moderate	1	3.13	1	3.13	2	6.30
Severe	6	18.8	10	31.3	17	53.13
CTQ physical abuse						
None	4	12.50	8	25.0	12	37.50
Low	2	6.30	4	12.50	6	18.80
Moderate	0	0	1	3.13	1	3.13
Severe	4	12.50	8	25.0	13	40.63
CTQ emotional abuse						
None	2	6.30	7	21.90	9	28.13
Low	2	6.30	6	18.75	8	25.00
Moderate	1	3.13	2	6.30	4	12.50
Severe	5	15.63	6	18.75	11	34.40
CTQ physical neglect						
None	3	9.40	7	21.90	10	31.30
Low	0	0	3	9.40	3	9.40
Moderate	2	6.30	5	15.63	8	25.00
Severe	5	15.63	6	18.75	11	34.40
CTQ emotional neglect						
None	3	9.40	8	25.0	12	37.5
Low	3	9.40	6	18.75	9	28.13
Moderate	2	6.30	2	6.30	4	12.50
Severe	2	6.30	5	15.63	7	21.90

Participants self-reported severity of childhood abuse and neglect using the Childhood Trauma-Questionnaire (CTQ).

### 3.3 Substance use

In the Sociodemographic and Life Circumstances Questionnaire, among the 32 participants who provided a response, 23 (74.20%) reported current substance use. Many participants experienced early exposure to substances (see Table 3), most commonly from family members, however, peer influence was also reported. Early exposure may be associated with premature onset of substance use, with this sample reporting a mean age of 15.63 (*SD* = 4.94, *Median* = 15.50, *Mode* = 12.00, *N* = 32) years old for their age of initiation (with the age range being six to 30 years).

**Table 3:** Participants living with HIV and substance use pattern.

Variable	Female (*n* = 10)	Male (*n* = 21)	Total sample (*n* = 32)
	*n* (%)	*n* (%)	*n* (%)
Current substance use (*n* = 31)			
Using	6 (19.35%)	17 (54.84%)	23 (74.20%)
Not using	4 (12.90%)	4 (12.90%)	8 (25.81%)
Age substance use initiation (*n* = 32)			
6–15	6 (18.75%)	10 (31.25%)	16 (50%)
16–22	2 (6.25%)	10 (31.25%)	13 (40.63%)
23–30	1 (3.13%)	2 (6.25%)	3 (9.38%)

Unequal sample sizes are due to one participant choosing not to specify their sex. The difference from the total sample size is due to an additional participant completing this survey.

### 3.4 Qualitative themes

Four main mental-health related themes were extracted from the semi-structured interview data for further analysis and interpretation: experiences of hardship, resilience, substance use as coping, and empowerment (Figure 1). Table 4 reports the most representative quotes by the main themes and sub-themes.

**Table 4:** Most representative quotes from participants living with HIV.

Subtheme	Quote #	Quote	Participant age and gender
Experiencing Hardships			
Childhood trauma	1	“My dad is an alcoholic, but he used to beat me all the time … But I grew up getting my ass kicked. So, like I’ve seen the bad side, but I’ve also seen the love.”	36, Man
	2	“Kids will mimic what you do, and kids will always remember right so the stuff I put my two oldest through is like (.) it still hurts to think about it.”	32, Woman
Separation of family	3	“Never stayed in one household more than three years growing up. So, I was like, in and out of CFS parent, to parent, family member to family member. And I’m the oldest daughter of my siblings.”	29, Woman
	4	“They got apprehended. And just like they went to daycare and some stranger pick[ed] them up. Like it was … And then after that I stayed sober for two months until I finally was like, I cannot do this. Like, I cannot frickin do this. So, I just started drinking again.”	32, Woman
Interpersonal violence	5	“I told the wrong person, which was my baby dad. And he was also abusive, too. So, he used to scream it out to … I would go drop my son off and then I’d have people drive me and he’d go banging on the window and just [say] she is HIV, you know she has HIV and just scream it. So, everyone basically found out after” … “He was really upset because I left him, he did not want me to leave I guess.”	34, Woman
HIV Stigma	6	“And there was a bit of prejudice there on [the doctor’s] part, but oh, yeah. I’ve felt that many, many, many times, with doctors, nurses, specialists, dentists, you name it. And at that time, nobody, like nobody wanted to touch you with a 10-foot pole because they were afraid they were going to catch it by being in the same room.”	63, Man
Racism	7	“Your race, or because a lot of people just think native people are gross, dirty alcoholics that do not give a shit about their life. But take a minute to sit down with someone, listen to their story. And a lot of them come from trauma.”	32, Woman
Resilience			
Health promoting cognitive processes	8	“Throughout my whole life, I was suicidal using drugs and drinking. And I’ve heard some people like, say, well, maybe your life will turn around for the better. I’ve heard other people’s lives that when they caught HIV, they turned their life around.”	29, Woman
	9	“This is the face of an HIV patient. And I want to tell you that this is not a death sentence for us. And there’s no need to be ashamed or, so this thing is. So that’s the very hard thing. It’s not about dealing [with] our condition.”	37, Woman
Healthy behavioural practices	10	“I’m undetectable. (.) I’ve been on it. I’ve been on the prog[ram] like I got with the program as soon as I found out. Never strayed.”	48, Woman
	11	[Motivated to join a detox program] “To not be struggling not to be homeless not to not to feel stuck in an area of my life that would never change.”	29, Woman
Enlisting social support from others	12	“And honestly, the care I have received from Nine Circles [HIV clinic] is amazing … This is, this place is part of my support circle, like it’s good … It’s just an accepting, loving environment … There’s no judgment here. No judgement at all. They, they understand everything. You know, it’s a lot more than others, I guess… Just like I said, just having an extended support circle of like family, like I would not even call them friends, they are more like family because they know my story.”	32, Woman
	13	“Only here is where I got help. If I wasn’t for this place [the HIV clinics], I would probably be dead right now. I really do not know where I would be.”	56, Man
Substance Use as Coping			
Reasons for substance use	14	“I started drinking because I was getting raped whenever I was younger, so that was pretty bad. And I started smoking crack because I was forced into it. I was choked out. Almost blacked out until I smoked it. And then, and then they would choke me again and smoking and they did that all night long. Till I was asking them for it. And then they said, okay, now you can start paying for [it]. So that’s how I got addicted to crack.”	34, Woman
	15	“Because when I first caught HIV was, I was homeless. And I was using drugs to numb the reality.”	29, Woman
	16	“I lost my place in the same month. I started injecting drugs.”	36, Man
Acquisition of substances	17	“I just … have addictions, like, you know, like, a lot of my days are based around what I do get like, it used to be just actually getting high all the time. But like nowadays, it’s not my first priority.”	33, Woman
Empowerment			
HIV acceptance	18	“I just remembers trynna to accept it then I would like, okay, like, trying to focus on what to feel, how I’m gonna feel. And then how to live life with it (.).”	29, Woman
	19	“As much as I was an addict, it was very helpful to know there was medication I was going to take that was going to help bring my viral load down. I tried my best to meet appointments, make appointments, and I did, and I made my appointments.”	32, Woman
Empowering other PLHIV and PWID	20	“Because it’s not a doctor … it’s not somebody who’s just clinical tech to tell you all the data and everything else. But if it’s somebody that’s taking the medications, lives it, deals with the side effects and all this. There’s a lot of little tricks in dealing with it that most people do not [know].”	63, Man

#### 3.4.1 Theme 1: experiencing hardships

Qualitative interviews corroborated the findings from the self-report questionnaires, as they revealed that most participants had undergone various hardships throughout their life, including childhood trauma, separation of family members, HIV stigma, interpersonal violence, and racism (Table 4; Quotes 1–7). Importantly, many participants spontaneously reported that they did not believe they had adequate support to deal with their unresolved trauma experiences.

##### 3.4.1.1 Childhood trauma

Several participants discussed experiences of childhood abuse and neglect, primarily from caregivers. Furthermore, some participants noted that being under the influence of drugs and/or alcohol hindered their caregiver’s ability to provide adequate care (Table 4; Quote 1). Some participants noted emulating coping strategies from caregivers once they began experiencing their own hardships. Some participants reported that an absence of a nurturing parental figure in their childhood hindered their ability to perform in those roles later (Table 4; Quote 2). Importantly, several participants reported that their experiences of trauma reduced their likelihood of seeking health care services, demonstrating that experiences of trauma can undermine patient trust.

##### 3.4.1.2 Separation of family members

Some participants discussed their involvement with the provincial child-care agency, Child and Family Services (CFS), as both children and parents. Several participants reported that CFS involvement in childhood was highly distressing, resulting in temporary, unstable living conditions (Table 4; Quote 3). Some women who were interviewed who had their children apprehended described this as a significantly traumatic event (Table 4; Quote 4). Several reported that the grief of their child’s removal resulted in turning to substance use. Conversely, some participants reported their child’s removal as a source of motivation and drive to improve their situation and mental health struggles, with the goal of proving they were fit to regain custody.

##### 3.4.1.3 Interpersonal violence

Several participants revealed that they were survivors of interpersonal violence, with women predominantly reporting previous or current involvement in abusive intimate relationships. Some of these women reported that the power imbalance in such relationships resulted in their partner preventing them from receiving and/or adhering to their HIV care, thereby weaponizing their HIV status as a tool for devaluation and isolation. Several women indicated that they believed that their intimate partners would capitalize on the communal stigma of HIV and disclose their HIV status, thereby preventing them from engaging in sexual relations with others. For example, a 34 year-old Indigenous woman, shared that she was in an abusive relationship with her child’s father. She reported that when he discovered she was diagnosed with HIV, he began tormenting her, ensuring those in their social circle were aware of her diagnosis. She stated that she felt ashamed and regretted revealing her HIV status to him, explaining “I told the wrong person, which was my baby *[sic]* dad. And he was also abusive, too. So, he used to scream it out to everyone like even when we broke up, he screamed at me… he’d go banging on the window and just [say] ‘she is HIV, you know she has HIV’ and just scream it” (Table 4; Quote 5).

##### 3.4.1.4 HIV stigma

Many participants reported facing HIV-related stigma, with the most common perpetrators being health care providers, friends, and family (Table 4; Quote 6). In the context of health care, participants reported that they experienced stigma from their general practitioners (e.g., family physicians, nurse practitioners) or during emergency room visits, especially when their HIV diagnosis occurred within the context of substance use dependence and precarious housing. Fear of being stigmatized resulted in many participants reporting that they concealed their HIV status from their community, to protect their social standing, and to prevent themselves and their children from discrimination.

##### 3.4.1.5 Racism

Four Indigenous participants reported that they had experienced differential treatment from service providers and attributed it to their race (Table 4; Quote 7). These individuals suggested that their Indigenous identity impacted the service they received, often due to lack of sensitivity or patience from health care providers.

#### 3.4.2 Theme 2: resilience

Despite experiencing various traumatic events, participants demonstrated resilience.

##### 3.4.2.1 Intrapersonal resilience

Some participants reported that they engaged in health-promoting cognitive processes, defined in the literature as gaining a sense of control, rationalizing the situation, and/or working towards improved health outcomes (6) (Table 4; Quotes 8–11). While receiving an HIV diagnosis was characterized as a traumatic event for many, it also presented an opportunity for change (Table 4; Quote 8). Many participants exhibited attempts to positively reframe their diagnosis and protest narratives such as HIV being a “death sentence” (Table 4; Quote 9). Participants also took the opportunity to highlight their own misconceptions about HIV prior to diagnosis, mainly attributing these to a lack of HIV education. Following their diagnosis, many participants reported that they wanted to learn more, with some taking the initiative to educate themselves about HIV. Several participants also indicated the importance of educating the public as a preventative measure, citing beliefs that this could have prevented their own acquisition of HIV.

A second form of intrapersonal resilience relates to participants’ engagement in healthy behaviours, such as ART adherence and attending medical appointments. Participants tended to take pride in attaining an undetectable viral load, highlighting the effort required (Table 4; Quote 10). Additionally, in both the interviews and in their self-report measure, participants discussed practicing safer sex (e.g., using condoms, pre- and post-exposure prophylaxis) and drug use (e.g., unused needles, using in safe environment). Most participants explained that since being diagnosed they have been making harm reduction-informed decisions, which were often not part of their previous routine. Furthermore, some participants reported that they had previously attended medical/social detoxification programs; many of these participants voiced that their motivations to do so were health-related. For example, a 29 year-old Indigenous woman explained that she previously admitted herself to detox, as she no longer wanted “to be struggling.” She also indicated her wish to “not be homeless, not to feel stuck in an area of my life that would never change” (Table 4; Quote 11).

##### 3.4.2.2 Interpersonal resilience

Regarding interpersonal modes of resilience, we examined the subtheme of *enlisting social support from others*, which emerged as the most-used resilience strategy (Table 4; quotes 12, 13). Some participants concealed their HIV status to avoid stigma, whereas several others reported receiving support from their family and friends. Of the participants who had personal support networks, many highlighted how unwavering support and encouragement were necessary for their well-being, with many highlighting the importance of receiving support for attending appointments and adhering to treatment.

Notably, across most interviews, participants described their HIV service providers as their greatest sources of support. Participants characterized the employees of HIV clinics as being instrumental to their HIV journey, with many recognizing the clinics as fostering trust and community. The support received from these service providers was cherished by participants who previously had been met with discrimination due to their HIV status. Some of the participants reported that when they had received their diagnosis in general health care settings they were met with stigmatized responses, but when transferred to a specialized HIV clinic they felt that the staff and the overall environment of HIV clinics were welcoming, non-judgmental and understanding (Table 4; Quotes 12, 13).

#### 3.4.3 Theme 3: substance use as coping

Regarding substance use, many participants provided insights into the functionality of their current use. For some individuals, a traumatic experience led to significant distress and, in some cases, mental illness (Table 4; Quote 14); substance use was considered a way to cope, especially in cases where substances were commonly used in their communities. Moreover, many participants who reported partaking in substance use earlier in life reported ongoing use. Beyond childhood experiences, reasons for individuals currently using were often tied to an event or persistent adverse circumstances, such as losing a loved one, interpersonal violence, and precarious housing (Table 4; Quotes 15, 16). Many participants also highlighted that preoccupation with acquiring substances prevented them from engaging with care (Table 4; Quote 17).

#### 3.4.4 Theme 4: empowerment

Throughout the qualitative interviews, participants reported ways they have gained an increasing sense of *empowerment* throughout their healthcare journey.

##### 3.4.4.1 HIV acceptance

When participants initially grappled with their newfound diagnosis, many reported feeling powerless. However, with increased HIV-related knowledge, many began to accept their HIV diagnosis; as such, they engaged in the *process* of empowerment (Table 4; Quote 18). Participants who accepted their diagnosis tended to report increased knowledge about their own condition (e.g., their current viral load), whether due to their own initiatives, accessing educational material, and/or through interaction with HIV service providers. Several participants emphasized the importance of their providers helping them participate in and guide their own health care choices (Table 4; Quote 19); it is possible that the acceptance received from these providers may have facilitated their own self-acceptance. Participants further noted that engaging in their own care allowed them to vocalize their concerns and gain knowledge, ultimately translating to the *outcome* of increased empowerment.

##### 3.4.4.2 Empowering other PLHIV and PWID

Some participants described how they were involved in the education of others on harm reduction, HIV, and other STBBIs. These participants explained that by empowering others they also empowered themselves. Some of the underlying motivations for educating others were revealed through participants who, while not currently involved in mentoring others, had voiced a desire to do so. For example, some participants explained that they wished to have been connected with a peer living with HIV at the time of their diagnosis and to provide the same support to others, as this would have allowed them to foster relationships with PLHIV and could have instilled a sense of optimism about their diagnosis. One male participant, who is 63 and white explained the value of receiving insights from a person “that’s taking the medications, lives it, [and] deals with the side effects,” as they can provide a different perspective than persons not living with HIV (Table 4; Quote 20). In addition, participants frequently shared lived and living experiences related to substance use and precarious housing, and that shared experience increased the credibility of peers/mentors. Some participants also self-identified as advocates wanting to encourage and support those who could not do so themselves.

## 4 Discussion

This study explores the mental health concerns and experiences of trauma faced by a sample of women and men living with HIV in Manitoba. To this end, we re-analyzed qualitative interviews about barriers and facilitators to HIV care, as well as self-report questionnaires related to childhood trauma and empowerment to better understand the mental health needs of this population. Using an intersectional framework, we recognized that many factors (e.g., gender, poverty, and race) influenced the health and well-being of our population, with some characteristics being associated with further marginalization, particularly when they occur simultaneously.

As commonly seen among PLHIV in the U.S., nearly 85% of our participants reported experiencing at least one form of childhood trauma (23). Our sample’s score on the CTQ-SF reflects moderate–severe experiences of childhood trauma and falls one point short from the severe-extreme category (24). MacDonald et al. (24) examined CTQ-SF scores in a large, international sample of nearly 20,000 participants, comprising community-based and psychiatric participants. In their study, 6% of community and 13% of clinical participants scored in the severe range, indicating high childhood trauma severity; in our sample, approximately 44% scored in the severe range, which is over triple that of their sample’s psychiatric patients (24). This raises the question of whether a relationship between childhood trauma and HIV risk exists, requiring further exploration. A study on PLHIV in South Africa found associations between childhood trauma and current HIV-risk behaviors (25), with the strength of this relationship moderated by trauma severity and frequency. The authors explained that the severity and frequency of childhood trauma had similar associations with HIV-risk behaviors for both women and men, however, more men reported high HIV-risk behaviors. Future research is needed on the relationship between childhood trauma and HIV-related outcomes (e.g., viral suppression) as some studies have identified a significant negative relationship between the two, even when controlling for confounders (26), whereas others have not (5). While the relationship between childhood trauma and HIV outcomes remains unclear, the experience of childhood trauma may also indirectly affect HIV-related outcomes through associations with other social determinants of health, especially in cases where the person exhibits post-traumatic stress symptoms (27). Further research is needed to identify early-life interventions that could prevent potential adult manifestations of childhood trauma.

In addition to childhood trauma, all participants reported experiencing at least one of several hardships throughout their life (e.g., familial separation, HIV stigma), with a significant number of participants having compounded experiences. Consequently, participants who had experienced more severe or multiple forms of hardships were those facing the greatest obstacles to engaging in health care. Of interest to us were how these hardships differed by sex and gender. Qualitatively, we noted that men tended to spontaneously report experiences of peer-related physical violence, with many of these events stemming from substance use, whereas women tended to spontaneously report current or previous experiences of intimate partner violence and familial separation.

Despite familial separation appearing as a prominent theme herein, minimal research has studied its impact on HIV outcomes. Studies have shown familial support can be instrumental to a person’s HIV journey (6, 28), therefore it is reasonable to consider that family separation may have negative impacts on HIV care. Interpersonal violence, which was reported by several participants, has also been found to negatively impact ART adherence and viral suppression (29). Studies have replicated this effect in cases of both intimate partner violence and cumulative forms of victimization, including verbal, physical, sexual, and/or witnessed violence (29). Participants also reported stigmatization due to their HIV status in health care and social settings; these findings are congruent with research highlighting the discrimination that PLHIV face due to their diagnosis, especially in cases where the individual also belongs to a racially marginalized group (11). The reduction of HIV-related stigma is crucial for increasing successful health outcomes for PLHIV, and is further supported by the United Nations’ Sustainable Development Goal 10: *reducing inequality* (30). This aim is important as exposure to stigma has been tied to reduced ART adherence, engagement, and retention in care along with greater HIV-related stress (31). Moreover, UNAIDS has underscored that experiences of discrimination and stigma may heighten risks of violence and marginalization (32). Therefore, to effectively address the disease burden associated with HIV, it is essential to tackle the socio demographic-related inequalities associated with HIV (33).

Hardships experienced by PLHIV can evolve into acute distress and/or chronic mental health conditions such as depression, which may exacerbate HIV symptoms (5). The fact that most participants experienced significant hardships underscores the importance of trauma-informed care and evidence-based mental health care for PLHIV, especially as these interventions hold promise for improved treatment outcomes (12). Remarkably, a few participants had voiced having experienced posttraumatic stress symptoms (i.e., distressing intrusion symptoms), even though these questions were not part of the interview. These experiences highlight the need for continued research on and increased mental health services for PLHIV who have experienced trauma, including intergenerational trauma (34).

The dearth of adequate mental health supports reported by participants likely contributes to the high frequency of current substance use, as several reported using substances to cope. Substance use behaviors including methamphetamine use in PLHIV have been associated with negative health outcomes including impediment of engagement in care, treatment nonadherence (35), weakened immune function, HIV progression, and increased risk of transmission (36). Additionally, substance use behaviors have been linked to poorer mental health outcomes for PLHIV (5). In our study, a significant portion of participants highlighted childhood onset of substance use, with one participant reporting using substances at age six. Early age onset is associated with substance use in adulthood, especially in the context of unresolved trauma (27). An increased understanding of the etiology and maintenance of substance use in the context of HIV is needed to improve HIV outcomes.

Traumatic experiences in the context of social, structural, and intergenerational factors culminate in an increased likelihood of initiating substance use. Alexander’s “Dislocation Theory of Addiction” provides insights for potential reasons why, in our study, participants displayed a high frequency and early initiation of substance use (37). Alexander explains that ‘addiction’ emerges from individual and social ‘dislocation,’ especially in cases of severe social, economic, and cultural dislocation. He posits that with time, these experiences result in shame and ostracization leading to discomfort; therefore, substance use as a remedy is both “functional” and “adaptive”.

Despite most participants having experienced several hardships, many displayed resilience and empowerment. Although there is some conceptual overlap between resilience and empowerment, we chose to parse their differences, as they do not always co-occur. Participants displayed resilience in three primary ways: engaging in adaptive cognitive processes, demonstrating effective health behaviors, and enlisting social support (6). Regarding intrapersonal forms of resilience, participants displayed resilience through health-promoting cognitive processes, with many reframing their HIV diagnosis to improve their outlook on their situation. Literature on cognitive processes in people living with chronic illness found that employing strategies reframing the meaning of a situation, spirituality, and forming support networks are linked to improved mental health (6). Engagement in healthy behavioural practices, including safer sex and drug use practices, along with increased exercise and improved diet were found to promote resilience in PLHIV (6, 28).

Regarding interpersonal forms of resilience, enlisting social support was most reported. To overcome hardships, several participants highlighted the importance of leaning on others for support, with the main sources discussed being participants’ family and HIV care providers. Many participants attributed their consistency with ART treatment to their family’s encouragement. These findings are congruent with literature highlighting the benefits of familial support to PLHIV’s well-being (6, 28). We also noted the frequent mention of the role of service providers as a facilitator for HIV care. Indeed, many participants highlighted how the HIV clinics were of the few places in which they felt comfortable revealing their HIV status. With time, participants built rapport with the staff and sometimes confided in them about their personal lives. A study conducted with specialized HIV health workers and their patients in Ghana found that the quality of patient-provider relationships, having welcoming environments, and provider’s positive perceptions of clients were crucial for reducing stigma and increasing engagement (38). These findings highlight the importance of improving health care provider attitudes to improve the experiences of PLHIV in primary and urgent care settings.

In our study, we identified two empowerment-related subthemes: HIV acceptance and empowering other PLHIV. Several participants in our sample displayed acceptance of their HIV diagnosis. Studies have shown that accepting one’s own medical condition in individuals living with chronic illnesses along with their increased involvement in care provides immense empowerment (8). Participants also reported engaging in the empowerment of others by providing advice, education, and/or support. This behavior can empower PLHIV as it provides purpose and fosters connection with others (6, 28). Importantly, this increased sense of empowerment can translate into improved treatment adherence (8).

### 4.1 Limitations

Our sample may not be representative of other PLHIV across Manitoba. Our data collection was limited to Winnipeg and Brandon, Manitoba (~85–90% of participants living with HIV live in these cities); it is possible that participants from these urban centers exhibit different mental health experiences compared to women, men and gender diverse individuals who reside in rural settings. Moreover, this study employed self-report quantitative measures in addition to qualitative interviews, as such, in all research that uses self-report measures there may be risk of bias (e.g., social desirability bias).

## 5 Conclusion

This qualitative study explored the experiences of PLHIV in Manitoba to better understand the relationship between mental health concerns and HIV treatment adherence. Participants reported a wide range of hardships, including adverse childhood experiences particularly sexual abuse), interpersonal violence (HIV stigma, racism, and socioeconomic-related stressors (e.g., houselessness, poverty). These experiences likely synergistically exacerbate participants’ current mental health concerns, leading to decreased HIV treatment engagement. However, participants also emphasized myriad ways they have demonstrated resilience and found empowerment in the context of their HIV diagnosis. To achieve the UNAIDS 95–95-95 targets by 2030, it is essential to understand the strengths and challenges of underserved populations, particularly the strategies that foster resilience, empowerment, and improved mental health outcomes for PLHIV in Manitoba.

## Data Availability

The original contributions presented in the study are included in the article/supplementary material, further inquiries can be directed to the corresponding authors.
